# Hippocampal‐striatal functional connectivity supports processing of temporal expectations from associative memory

**DOI:** 10.1002/hipo.23205

**Published:** 2020-04-10

**Authors:** Vincent van de Ven, Chanju Lee, Julia Lifanov, Sarah Kochs, Henk Jansma, Peter De Weerd

**Affiliations:** ^1^ Department of Cognitive Neuroscience, Faculty of Psychology and Neuroscience Maastricht University Maastricht The Netherlands; ^2^ University of Birmingham Birmingham UK

**Keywords:** associative memory, functional connectivity, hippocampus, striatum, temporal context

## Abstract

The hippocampus and dorsal striatum are both associated with temporal processing, but they are thought to play distinct roles. The hippocampus has been reported to contribute to storing temporal structure of events in memory, whereas the striatum contributes to temporal motor preparation and reward anticipation. Here, we asked whether the striatum cooperates with the hippocampus in processing the temporal context of memorized visual associations. In our task, participants were trained to implicitly form temporal expectations for one of two possible time intervals associated to specific cue‐target associations, and subsequently were scanned using ultra‐high‐field 7T functional magnetic resonance imaging. During scanning, learned temporal expectations could be violated when the pairs were presented at either the associated or not‐associated time intervals. When temporal expectations were met during testing trials, activity in left and right hippocampal subfields and right putamen decreased, compared to when temporal expectations were not met. Further, psycho‐physiological interactions showed that functional connectivity between left hippocampal subfields and caudate decreased when temporal expectations were not met. Our results indicate that the hippocampus and striatum cooperate to process implicit temporal expectation from mnemonic associations. Our findings provide further support for a hippocampal‐striatal network in temporal associative processing.

## 1 INTRODUCTION

Extensive research on episodic memory has supported the suggestion that the ability to correctly order events into a coherent and continuous sequence (Kurby & Zacks, 2008; Tulving, 1984) is crucial for various cognitive abilities and functioning of our daily life (e.g., [Vargha‐Khadem et al., 1997; Bartsch, Dohring, Rohr, Jansen, & Deuschl, 2011; Baker, Dexter, Hardwicke, Goldstone, & Kourtzi, 2014]). A growing body of literature supports the suggestion that temporal context can facilitate memory processes, such as enhancing encoding of events when they are experienced rhythmically (Jones & Ward, 2019; ten Oever & Sack, 2019), as well as facilitating memory retrieval when items are shown in the same temporal context as during initial encoding (Cravo, Gohenkohl, Santos, & Nobre, 2017; Thavabalasingam, O'Neil, Zeng, & Lee, 2016; van de Ven, Kochs, Smulders, & De Weerd, 2017). It is possible that temporal contexts may serve to bind discontiguous events in memory and provide a means to form expectations from memory about what will happen in the near future. In parallel, there is rapidly growing consensus from both animal neurophysiological and human neuroimaging research that the medial temporal lobe, including the hippocampus, is involved in representing temporal information in memory (Eichenbaum, 2013, 2014; Ranganath & Hsieh, 2016). Yet, processing of temporal information has long been associated with activity in the dorsal striatum (DS) and other parts of the motor circuit (Matell, Meck, & Nicolelis, 2003; Meck, Penney, & Pouthas, 2008; Mello, Soares, & Paton, 2015). Further, the DS and hippocampus can show increased functional connectivity during memory encoding or retrieval in spatial associative contexts (Voermans et al., 2004; Woolley et al., 2015). Investigation of hippocampal‐striatal interaction during temporal associative contexts has not yet been described. This was the aim of the current study.

The medial temporal lobe, specifically the hippocampus, has been suggested as a primary brain region for processing spatial navigation and episodic memory (Bird & Burgess, 2008; Milner, Squire, & Kandel, 1998; Squire, 1992). Over the last decade, researchers reported that time is also processed in the hippocampus (Eichenbaum, 2014). Single cell recordings in the rat hippocampus found peak firing of hippocampal cells at successive moments during delay periods inserted between cue and probe within trials of a paired associate task (MacDonald, Carrow, Place, & Eichenbaum, 2013; MacDonald, Lepage, Eden, & Eichenbaum, 2011; Pastalkova, Itskov, Amarasingham, & Buzsáki, 2008). The activities of these so‐called “time cells” might reflect encoding of the temporal dimensions of events, a crucial property of episodic memory (Ergorul & Eichenbaum, 2004; Tulving, 1984). This argument was further supported by lesion studies with rats that showed hippocampal damage results in disruption of memory for time without impairing the recognition of items in learned sequences (DeVito & Eichenbaum, 2011; Ergorul & Eichenbaum, 2004). Further, many of these studies suggest that time cells are largely represented in specific hippocampal subfields, most notably CA3–CA1 (Farovik, Dupont, & Eichenbaum, 2010; MacDonald et al., 2011; Mankin, Diehl, Sparks, Leutgeb, & Leutgeb, 2015), although there is some evidence for the involvement of dentate gyrus (DG) in mnemonic coding of time (Aimone, Wiles, & Gage, 2006; Rangel et al., 2014).

Similar to rodent research, human neuroimaging research also observed activity in the hippocampus that is compatible with temporal associative memory. A number of studies showed differential activations in the hippocampus during tasks in which temporal contexts changed (e.g., [Staresina & Davachi, 2009; Schapiro, Kustner, & Turk‐browne, 2012; Ezzyat & Davachi, 2014; Thavabalasingam, O'Neil, & Lee, 2018; Thavabalasingam, O'Neil, Tay, Nestor, & Lee, 2019]). Importantly, the hippocampus seemed to be mostly related to bridging the temporal gap between objects that were presented sequentially and in close temporal proximity. For example, one study investigated the effects of temporal order of memorized sequences of objects (Ezzyat & Davachi, 2014), and found that hippocampal patterns were more similar for objects when their positions were temporally close within a learned sequence. Thus, rather than a clock, the hippocampus might function as an associator of information across different moments in time. In further support of this notion are findings that suggest that the hippocampus may encode relative temporal structure at different time scales (Mankin et al., 2015).

In parallel lines of research, the DS, including caudate nucleus and putamen, has long been strongly associated with temporal processing of stimulus‐response events (Buhusi & Meck, 2005; Coull, Cheng, & Meck, 2010; van Rijn, Gu, & Meck, 2014). DS neurons have been found to increase activity when a behaviorally relevant period of time is about to be over (Matell et al., 2003; Mello et al., 2015). Further, DS lesions in rats (Meck, 2006), neurological diseases affecting dorsal striatal areas (Malapani et al., 1998), and disruptions in DS dopamine signaling (Rowe et al., 2010), interfere with the processing of durations. In addition to the findings from rodent research, human neuroimaging studies also reported higher striatal activity when participants were engaged in tasks that required the processing of interval timings (Coull, Vidal, Nazarian, & Macar, 2004; Ferrandez et al., 2003; Rao, Mayer, & Harrington, 2001; Tanaka et al., 2004). The majority of these studies focused on motor preparation to timed or rhythmic responses, with little to no investigation of the possible relation to episodic memory formation. However, DS, particularly putamen, may also be involved in processing violations of temporal expectancy about reward delivery (Doherty et al., 2004; Mcclure, Berns, & Read Montague, 2003; Seymour et al., 2004). For example, Mcclure et al. (2003) used a classical conditioning paradigm in which participants either learned that a reward was delivered 6 s after cue onset or after an unpredictable interval. During the test phase, the reward in the previously predictable context could now on some trials unexpectedly be delivered 4 s later. Results showed increased left putamen activity for the unexpectedly delayed reward, compared to reward delivered at the predicted interval of 6 s, suggesting that DS processes temporal prediction errors that indicate violation of expectations.

Interestingly, the hippocampus has shown increased functional connectivity with DS during encoding of new episodic (Sadeh, Shohamy, Levy, Reggev, & Maril, 2011) or associative memories (Mattfeld & Stark, 2015). Moreover, both subcortical structures appear to be involved in spatial processing and navigation through a real or virtual environment (Gengler, Mallot, & Hölscher, 2005; Igloi, Doeller, Berthoz, Rondi‐Reig, & Burgess, 2010; Voermans et al., 2004), suggesting that they interact when processing the contextual aspects of associative events in memory encoding. Further, one study showed increased hippocampal‐striatal connectivity when detecting unexpected temporal durations (Barnett, O'Neil, Watson, & Lee, 2014). Whether this interaction also plays a role in associative memory of time has not yet been investigated.

The purpose of this study was to test whether associative temporal memory is related to hippocampal‐striatal connectivity in the human brain. To this end, we had participants learn cue‐target associative pairs of visual stimuli in different temporal contexts, in the form of different time intervals between a cue and target (van de Ven et al., 2017). An important property of these memories was that each cue was hypothesized to elicit neural responses representing the prediction of the temporal context in which the associated target event would follow in the near future, that is, when the associated target would appear. During memory testing, participants could be presented with cue‐target pairs in the learned as well as in a novel temporal context. This discrepancy of brain activity in different temporal contexts was measured using ultra‐high field (UHF) functional magnetic resonance imaging (fMRI) at 7 T. The main analyses focused on dorsal striatal structures and hippocampal subfields in the left and right hemispheres, in which we analyzed regional activity and interregional connectivity as a function of memory‐based temporal expectancy. We hypothesized that striatum and hippocampus would show higher activity when temporal expectancies from memory were not met. Further, we hypothesized decreased hippocampal‐striatal functional connectivity, as analyzed using psycho‐physiological interactions (PPI) when temporal expectancies were not met.

## 2 METHODS

### 2.1 Participants

Eighteen healthy young adults (mean [SD] age = 23.06 [3.02] years, 15 females) participated in the study. To ensure suitability with the MR environment, all participants were screened by experimenters before participation. All participants provided written informed consent to participate in the study and MR measurements, and received financial compensation for their participation. The study was approved by the local ethics committee of the Faculty of Psychology and Neuroscience (FPN) of Maastricht University.

### 2.2 Task procedure

Participants completed a time paired associative task (van de Ven et al., 2017) in which they learned to associate pairs of cue‐target stimuli, which were separated by one of two time delays. For the stimuli, we used eight pairs of abstract shapes to minimize conceptual processing and make the task challenging. Associated stimulus pairs were randomly created for each participant.

Participants first learned the cue‐target pairs outside the MR scanner, with the task presented on a laptop screen. During the testing phase, stimuli were delivered to participants at the same visual angles through a mirror system while lying in the MR scanner. Each stimulus was shown at a size of 5° × 5° visual angle at the center of the screen, on a grey surface. When no stimulus was presented, a fixation cross was presented at a size of 1° × 1° visual angle. The experiment was programmed in Psychopy version 1.8 (Peirce, 2007), using its feature of screen refresh readout (refresh rate = 60 Hz) to maximally control stimulus and interval timing (Garaizar & Vadillo, 2014).

**FIGURE 1:**
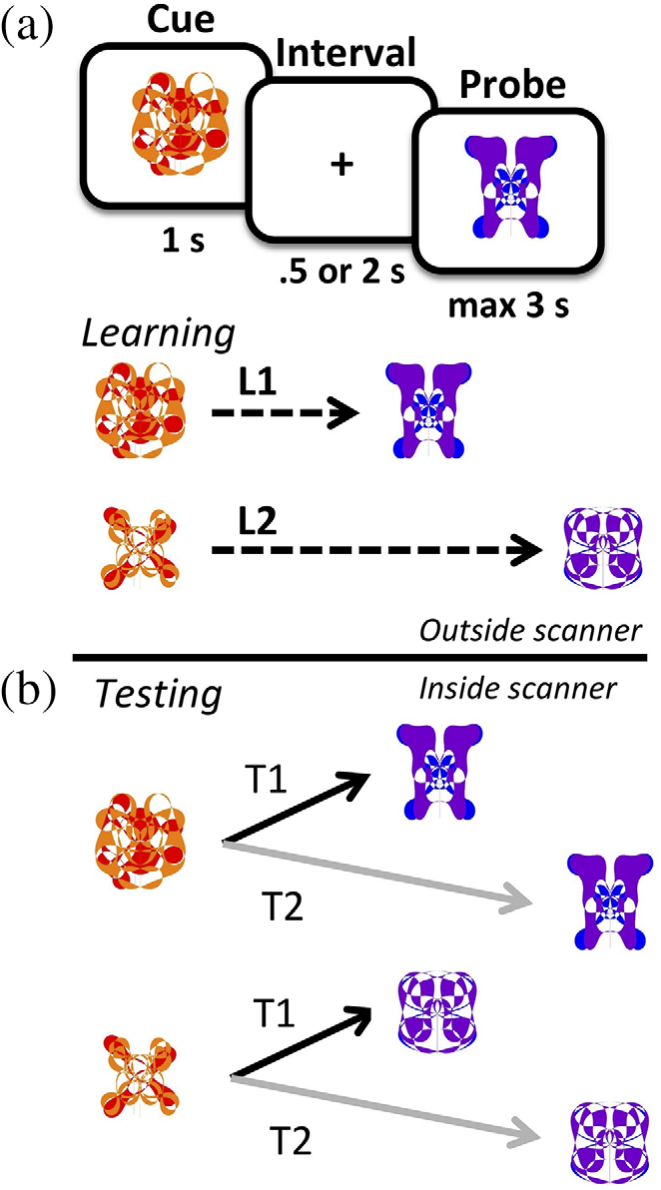
Task design. Participants had to learn cue‐target associations, in which items were presented sequentially. Each pair was further association with one of two time intervals, L1 (500 ms) or L2 (2000 ms). During memory testing, each cue‐target pair could be shown with either of the two intervals, T1 (500 ms) and T2 (2000 ms) [Color figure can be viewed at wileyonlinelibrary.com]

The task started with a learning phase (see Figure 1). At the start of the learning phase, the eight cue‐target pairs were shown to participants once and without the requirement to respond to the items (passive exposure trials). Each trial began with the presentation of a fixation cross (500 ms) after which the cue item was shown (1,000 ms). After cue offset, the target item was shown for 1,000 ms at a delay interval of either 500 or 2000 ms (respectively, L1 and L2 for short or long intervals during learning), with each pair assigned to one of the two intervals. Participants were not informed about the different temporal contexts. Participants then trained to learn and memorize the eight cue‐target pairs. Each trial was similar in design as for the passive exposure trials, with two important exceptions. First, the latter item (referred to as probe) of each presented pair could either be the associated target (as seen during the passive exposure trials) or one of the seven nontarget alternatives, randomly drawn on each nontarget trial. Second, probes that were targets were always shown after the associated interval. When probes were nontargets, the interval was always the nonassociated delay. Cues were shown with the associated targets (and thus with their associated intervals) in 50% of the trials. Participants had to determine whether the probe item was the cue‐associated target and indicated their decision through a button press response within 3,000 ms after probe onset. Response feedback (color change of the fixation cross indicating a correct [green] or incorrect [red] response) was provided after each trial to facilitate learning of the stimulus associations. One learning block consisted of 32 trials, which were presented in random order, and participants repeated learning blocks until reaching 84.38% accuracy (27 correct trials out of 32) within a learning block or until a maximum of 6 learning blocks were completed.

After the learning phase, participants underwent the testing phase in the MR scanner. The testing phase was similar to the learning phase, with two crucial differences. First, the pairing between cue and interval was broken, such that each cue was shown with either of the two intervals with equal probability of 0.5, regardless of whether the probe matched the target or not. We denote intervals between the cue and probe in the testing phase as T1 (500 ms) and T2 (2000 ms). Second, participants were not given any feedback about their responses throughout the testing phase. Trial order was randomized for each block and each participant. One testing block consisted of 64 trials and participants completed 2–3 testing blocks in the scanner. The intertrial interval for the testing phase was jittered around an average of 8,000 ms. After the session was completed, participants were asked whether they noticed any changes in temporal gaps between cue‐target stimuli. An entire testing session (inside and outside of the scanner) lasted approximately 90–120 min and concluded with debriefing.

### 2.3 MRI data acquisition

A Siemens MAGNETOM 7 Tesla MR scanner with a 32‐channel head coil was used to acquire whole‐brain imaging data. An EPI sequence was used to collect blood oxygenation level‐dependent (BOLD) images. All scanning sessions were held at the Scannexus facility in Maastricht, the Netherlands. Participants were instructed to fixate their head and posture throughout the scanning. Before collecting any images, semiautomated shimming was performed. After the shimming, anatomical data were acquired at two different inversion times (TI_1_ and TI_2_) within the same measurement (MP2RAGE, [Marques et al., 2010]; TI_1_/TI_2_ = 900/2750 ms, TR = 5 s, 0.7 mm isotropic, 240 slices, no interslice gap, acquired with fat suppression). T1 images were then calculated from the ratio of the contrast images of the two inversion times, which provides increased signal homogeneity at sub‐millimeter resolution. The MP2RAGE multi‐contrast images, at sub‐millimeter resolution, provide high potential for reliable segmentation of brain tissue and structures (Choi, Kawaguchi, Matsuoka, Kober, & Kida, 2019; Næss‐Schmidt et al., 2016). Functional images were collected using T2*‐weighted images (1.25 mm isotropic, 60 slices, no interslice gap, TR = 1.5 s, TE = 22 ms, FA = 50, anterior‐to‐posterior phase direction). Speed of data acquisition was increased using a multi‐band acquisition sequence of two simultaneous slices and a GRAPPA acceleration factor of 2 (Moeller et al., 2010). Additionally, five phase‐inverted (posterior‐to‐anterior) EPI images were collected with the same imaging parameters for offline geometric distortion correction (see below). While participants were lying inside the scanner, experimental stimuli were delivered through a mirror system. During the functional runs, behavioral responses were simultaneously collected using an MR‐compatible button box.

### 2.4 Analysis

#### 2.4.1 Preprocessing

Imaging data were preprocessed and analyzed using the BrainVoyager v20.4 (Goebel, Esposito, & Formisano, 2006) and the NeuroElf toolbox (http:/neuroelf.net) in MATLAB 2015a (www.mathworks.com). First, anatomical MR images were corrected for intensity inhomogeneity, skull‐stripped and then spatially normalized to the MNI (Montreal Neurological Institute)‐152 template space using an affine registration with 12 degrees of freedom. For functional images, processing steps included slice scan time correction (sinc interpolation), three‐dimensional (3D) motion correction and temporal filtering using linear trend removal and high‐pass filtering (4 sine/cosine cycles across the full timecourse). Geometrical distortions in functional images that resulted from EPI sequences at 7 T were corrected using a set of five phase‐inversed EPI baseline images with the Correction based on Opposite Encoding plugin version 1.0, which follows a previously published offline image correction approach (Andersson & Skare, 2002; Andersson, Skare, & Ashburner, 2003). Geometrically corrected and preprocessed functional images were then normalized to MNI space.

#### 2.4.2 Region‐of‐interest creation

**FIGURE 2:**
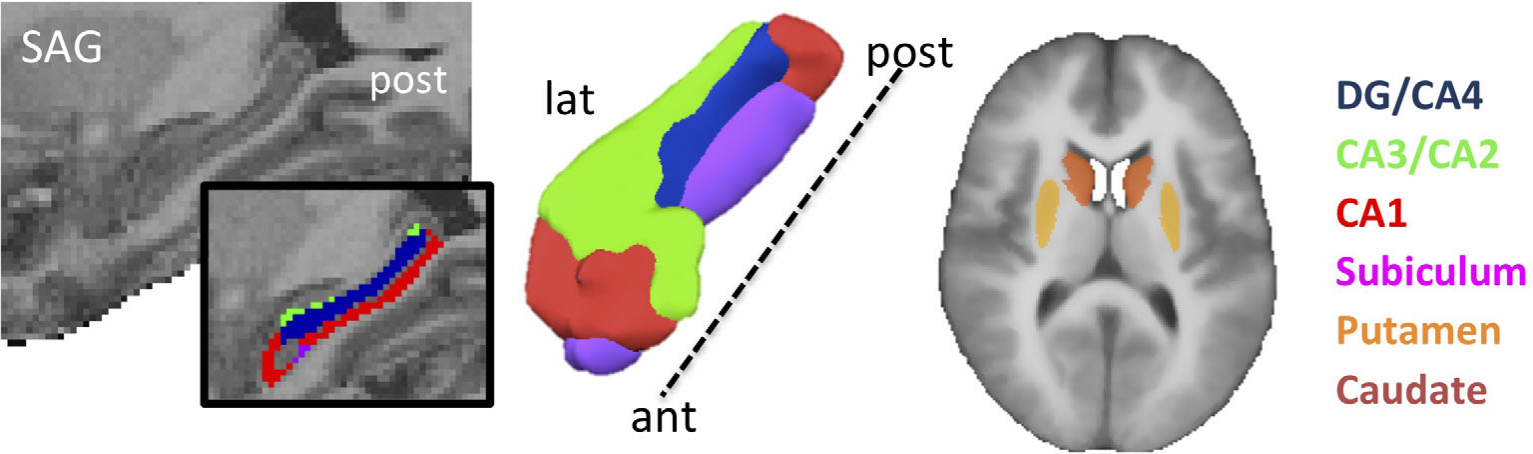
Hippocampal and dorsal striatal region of interest (ROIs). The hippocampal subfields of one participant are superimposed on the anatomical image of that participant. The striatal ROIs are superimposed on the average anatomy of all participants [Color figure can be viewed at wileyonlinelibrary.com]

For the purpose of this study, the hippocampus and striatum were a priori selected as regions‐of‐interests (ROIs). Bilateral hippocampus and its subfields were segmented from the T1 images of each participant individually using the online anatomical processing pipeline volBrain (http://volbrain.upv.es; Manjón & Coupé, 2016). It has been shown that volBrain can very reliably segment brain structures using high‐resolution MP2RAGE multi‐contrast images (Næss‐Schmidt et al., 2016). In this pipeline, hippocampus and its subfields are localized and extracted from each anatomical image using a patch‐based segmentation method (Coupé et al., 2011; Romero, Coupé, & Manjón, 2017), which resulted in four ROIs for hippocampal subfield DG/CA4 (DG/CA4), CA3/CA2, CA1 and Subiculum (Sub) in each hemisphere for each participant. Left and right striatal ROIs were taken from an anatomical atlas of the basal ganglia that was based on high‐resolution 7 T multi‐modal MR images in young adults (Keuken et al., 2014). We separated the dorsal striatal ROIs in each hemisphere into the caudate nucleus and putamen using NeuroElf and BrainVoyager's segmentation tools. Figure 2 depicts the hippocampal and striatal ROIs used in the study and Table 1 lists the ROI sizes in mm^3^ (averaged across participants for the hippocampal subfields).

**TABLE 1:** ROI sizes (*k*) in mm^3^

ROIs	Mean (*k*)	*SE* (k)
*Hippocampus* ^a^		
rDG/CA4	901.88	50.01
rCA3/CA2	148.50	10.19
rCA1	925.75	47.92
rSub	255.75	22.04
lDG/CA4	904.31	45.74
lCA3/CA2	128.56	10.81
lCA1	962.19	35.33
lSub	301.75	14.41
*Striatum* ^b^		
LCaudate	5,689.00	‐
RCaudate	6,864.00	‐
LPutamen	6,764.00	‐
RPutamen	7,326.00	‐

a The hippocampal subfields were estimated from volBrain segmentations of the anatomical images for each participant.

b Striatal structures were obtained from a 7T‐based atlas.

#### 2.4.3 Statistical analysis

Behavioral performance during the testing phase was analyzed using paired‐sample tests with sensitivity (*d*′) as dependent variable. Functional data were analyzed using a multi‐level general linear model (GLM) with the four interval‐based task conditions (L1×T1, L1×T2, L2×T1, and L2×T2) containing correctly responded trials only and two additional conditions for inaccurately responded trials or trials with missing responses. The full extent of each trial, including cue, delay and probe, was modeled as a single event, such that T2 predictors were longer in time than T1 predictors. GLM predictors were convolved with a two‐gamma hemodynamic response function (HRF). Note that, within each class of test trials, events originating from the different learning intervals L1 and L2 were modeled with the same length and amplitude after HRF deconvolution. The task regressors were appended with six (Z‐normalized) head motion displacement vectors, as estimated by BrainVoyager's head motion correction procedure (Christoffels, van de Ven, Waldorp, Formisano, & Schiller, 2011; Goebel et al., 2006), and their first derivates. The GLM was applied at the ROI level, at which timeseries of voxels within an ROI were sampled and averaged for each participant. At the first analysis level, the GLM was fit to the functional data of each participant. At the second level, the single‐subject GLM coefficients for the task conditions were analysed at the subject‐level using a Random Effects (RFX) approach. Particularly, we were mainly interested in long‐interval trials (that is, L1×T2 and L2×T2) as in these trials the interval was captured by at least one MR functional volume. Moreover, in the L1×T1 and L2×T1 trials the time interval between stimuli was short so that effects related to temporal expectation may be masked by signal related to the stimulus presentation. Thus, we focused on the contrast [L1×T2–L2×T2]. Statistical results were corrected for multiple comparisons (eight hippocampal and four striatal ROIs) using a false‐discovery rate (FDR) of *q* = 0.05 (Benjamini & Hochberg, 1995; Genovese, Lazar, & Nichols, 2002).

In addition, an explorative voxel‐by‐voxel analysis was performed at the whole‐brain level using the RFX GLM to explore activations that were induced by the task paradigm outside of the ROIs. Multiple comparison correction was performed at the cluster‐level, using a Monte Carlo simulation of 1,000 random statistical images of which values were drawn from a normal distribution and in which the spatial smoothness of each simulation was based on the empirical statistical map (Forman et al., 1995; Goebel et al., 2006). Clusters from the simulated maps were tabulated and ranked in size, from which the cluster size at a false positive rate of .05 was taken as minimum cluster threshold for visualizing the empirical map.

#### 2.4.4 Functional connectivity analysis: PPIs

Functional connectivity between ROIs engaged in our task paradigm was investigated using PPI analysis (Friston et al., 1997; O'Reilly, Woolrich, Behrens, Smith, & Johansen‐Berg, 2012). The PPI design matrix, including the interaction term, was generated for each participant separately using the NeuroElf toolbox. For each PPI, the psychological and physiological variables were deconvolved prior to calculating the interaction term, which was then convolved with a two‐gamma HRF. The PPI model was then tested in a similar two‐level RFX approach as the task‐based GLM, with regression fits estimated for each individual (first‐level) combined into a group‐level test of significance (second‐level).

## 3 RESULTS

### 3.1 Behavioral results

Data of two participants who failed to press response buttons within the maximum response duration on more than half of the trials were discarded, as well as the data of one participant with corrupted MR image files. We analyzed the behavioral and fMRI data of the remaining sample (*N* = 15).

During the learning phase, all participants reached learning criterion, completing 3.27 blocks on average (median = 3, *SE* = 0.27, range = 2–5 blocks). Maximum memory performance (*d*′) at the end of the learning phase was 2.88 on average (*SE* = 0.15).

We analyzed performance of the testing phase using a repeated measures ANOVA with Learn (L1, L2) and Test Intervals (T1, T2) as within‐subject factors. We found an effect for learn interval that approached significance (*F*_(1,14)_ = 4.58, *p* = .051), no significant effect for test intervals (*F*_(1,14)_ = 0.67, *p* = .43), and a significant interaction effect (*F*_(1,14)_ = 6.97, *p* = .019, $\varepsilon_{p}^{2}$=0.33). Posthoc comparisons showed that participants were better at judging whether the probe matched the cue when the T2 test interval matched the learned interval, L2 (mean [*SE*] *d*′ = 1.53 [0.35]), compared to when it did not match the learned interval, L1 (mean [*SE*] *d*′ = 0.68 [0.36]; *t*(14) = −3.93, *p* = .0013, Cohen's *d* = −0.93). For T1 trials, accuracy did not significantly differ between learning intervals L1 (1.06 [0.44]) and L2 (1.40 [0.36]; *t*(14) = −1.25, *p* = .23). Further, accuracy for L1 trials significantly decreased when shown during testing with the nonmatching interval (T2, 0.78 [0.43]) compared to the matching interval (T1, 1.20 [0.51]; *t*(14) = −2.26, *p* = .04, Cohen's *d* = −0.58). For L2 trials, this effect was in the same direction (T1, 1.37 [0.42]; T2, 1.59 [0.41]) but not significant (*p* = .19). Behavioral results were very similar when data of the participant with corrupted imaging data were included in this analysis.

Of note, it could be argued that the trend of lower *d*′ during retrieval for L1 compared to L2 trials could be an indication of generally weaker memory for L1 items. Importantly, we found no evidence for a performance difference between L1 and L2 pairs during each participant's last block of learning (*t*(14) = 0.04, *p* = .97). Furthermore, maximum overall performance after reaching learning criterion was not correlated to the interaction effect (*p* > .70) or to the learning interval difference in T2 trials (*p* > .40), thus limiting the possibility that a difference in memory strength during retrieval stemmed from memory encoding. To verify that a difference in memory strength did not explain our results, we correlated the L1 versus L2 contrast with the L1×T2 versus L2×T2 contrast at the subject‐level, and found no significant effect (*p* = .76). Together, these findings suggest that a possible difference in memory strength for L1 versus L2 items does not provide a strong alternative for our interpretation that the L1×T2 versus L2×T2 performance differences are due to a violation of temporal expectations.

### 3.2 ROI analyses: hippocampus and striatum

Activation statistics for each ROI per condition L1×T2 and L2×T2 against resting baseline are listed in Table 2. Hippocampal subfields generally showed significantly decreased activity when the temporal interval during retrieval matched the interval during learning (L2×T2). Dorsal striatal structures showed significantly increased activity for both conditions, although activity was higher when the temporal interval during retrieval did not match the interval during learning (L1×T2).

**TABLE 2:** Region of interest (ROI) results

	L1×T2					L2×T2				
ROIs	M	*SEM*	T	*p*	Cd	M	*SEM*	T	*p*	Cd
lDG/CA4	−0.11	0.06	−1.87	.084	−0.48	−0.24	0.06	−4.27	.001	−1.10*
lCA3/CA2	−0.10	0.04	−2.34	.035	−0.61	−0.23	0.04	−6.23	0	−1.61*
lCA1	−0.08	0.03	−2.58	.021	−0.67	−0.18	0.04	−4.93	0	−1.27*
lSub	−0.01	0.07	−0.13	.894	−0.03	−0.19	0.06	−3.14	.009	−0.81*
LCaudate	0.38	0.13	2.89	.002	0.75*	0.15	0.07	2.10	.054	0.54
LPutamen	0.17	0.11	1.61	.138	0.42	−0.08	0.07	−1.16	.269	−0.30
rDG/CA4	−0.04	0.07	−0.59	.554	−0.15	−0.16	0.05	−3.08	.011	−0.80*
rCA3/CA2	−0.10	0.05	−1.91	.078	−0.49	−0.13	0.04	−3.06	.013	−0.79*
rCA1	0.01	0.05	0.20	.847	0.05	−0.12	0.04	−2.78	.017	−0.72*
rSub	0.01	0.04	0.27	.791	0.07	−0.09	0.05	−1.94	.072	−0.50
RCaudate	0.35	0.13	2.72	.003	0.70*	0.19	0.09	2.19	.041	0.57
RPutamen	0.20	0.09	2.26	.03	0.59*	−0.03	0.07	−0.42	.686	−0.11

*Note*: Signal activation statistics for conditions L1×T2 and L2×T2 against resting baseline in each ROI (degrees of freedom = 14). *significant at false‐discovery rate *q* = 0.05.

Abbreviations: Cd, Cohen's *d*; DG, dentate gyrus; L/R, left/right; Sub, subiculum.

**FIGURE 3:**
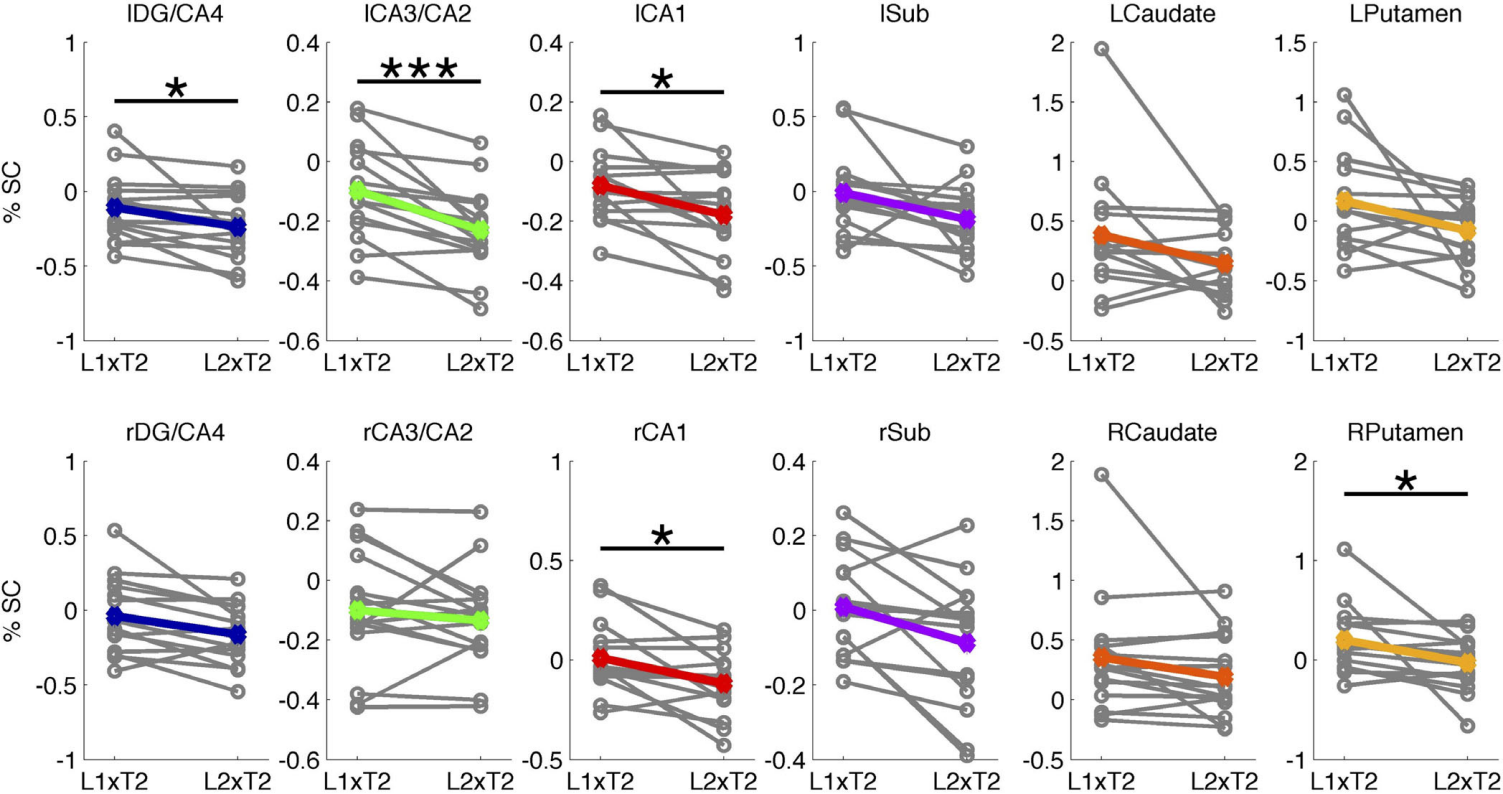
ROI results. Mean activity during conditions L1×T2 and L2×T2 for each of the hippocampal subfields and dorsal striatal nuclei. *p < .05, ***p < .005 (corrected for multiple comparisons) [Color figure can be viewed at wileyonlinelibrary.com]

Several ROIs showed significant differences in activity between the two conditions. Figure 3 shows the distribution of regression coefficients between the two conditions for the four hippocampal subfields and the two DS nuclei in each hemisphere. For hippocampal subfields, we found significant deactivations when intervals matched temporal expectations (L2×T2) compared to when they did not (L1×T2) in left hippocampal subfields (DG/CA4: *t*(14) = −2.62, *p* = .004, adjusted‐*p* = .039, Cohen's *d* = −0.68; CA3/CA2: (*t*(14) = −4.68, *p* < .001, adjusted‐*p* < .004, Cohen's *d* = −1.21; CA1: (*t*(14) = −3.02, *p* = .002, adjusted‐*p* = .030, Cohen's *d* = −0.78) as well as right CA1 (*t*(14) = −2.99, *p* = .007, adjusted‐*p* = .049, Cohen's *d* = −0.77). For all other hippocampal subfields, corrected *p*‐values were larger than .05.

For the dorsal striatal areas, we found a significant decrease in activation in right Putamen (*t*(14) = −2.34, *p* = .004, adjusted‐*p* = .039, Cohen's *d* = −0.61) when intervals matched expectations, compared to when they did not. Effects in left Putamen and the Caudate nuclei were not significant (corrected *p*s > .05).

When analyzing ROI activity for the short testing trials (i.e., L1×T1 vs. L2×T1) we found no significant differences in any of the ROIs (corrected *p*s > .34). This null finding corresponded to the absence of a significant behavioral effect for T1 trials, while at the same time suggesting that the behavioral and fMRI effects for T2 trials were related.

To further investigate the relation between brain activity and temporal expectation at the subject‐level, we correlated activity of left hippocampal subfields and right putamen with task performance pooled across both conditions. Given the difference in sign of activity in the two areas, we would expect that better task performance was associated with decreased CA1 activity, but with increased putamen activity. We found significant negative correlations (Spearman, corrected for multiple comparisons) between *d*′ and left DG/CA4 (rho = −0.44, *p* = .014, adjusted‐*p* = .048), left CA3/CA2 (rho = −0.43, *p* = .017, adjusted‐*p* = .048) and left CA1 (rho = −0.62, *p* < .001, adjusted‐*p* = .002). The correlation between *d*′ and right putamen was positive but not significant (rho = 0.30, *p* = .11).

### 3.3 PPI of the left hippocampus × task onto the striatum

In this analysis, we investigated whether functional connectivity between the left hippocampus and each of the four striatal nuclei changed with different task conditions. We applied a PPI model with the task contrast of L1×T2 versus L2×T2 as psychological factor and the average activity of the three left hippocampal subfields (DG/CA4, CA3/CA2, CA1) as physiological factor to each of the four ROIs of the DS. We found a significant PPI interaction term for left caudate (*t*(14) = −2.96, *p* = .011, adjusted‐*p* = .045, Cohen's *d* = −0.76). The PPI interaction term for right putamen was only significant at the uncorrected p‐threshold (*t*(14) = −2.26, *p* = .041, adjusted‐*p* = .12). To test whether each of the three hippocampal subfields was functionally connected to left caudate, we conducted additional separate PPIs with left caudate activity as dependent variable and activity in each of the three hippocampal subfields as physiological factor. All three hippocampal subfields showed significant functional coupling with the left caudate (DG/CA4: *t*(14) = −3.13, *p* = .0038, Cohen's *d* = −0.81; CA3/CA2: *t*(14) = −2.59, *p* = .019, Cohen's *d* = −0.69; CA1: *t*(14) = −2.45, *p* = .022, Cohen's *d* = −0.63).

**FIGURE 4:**
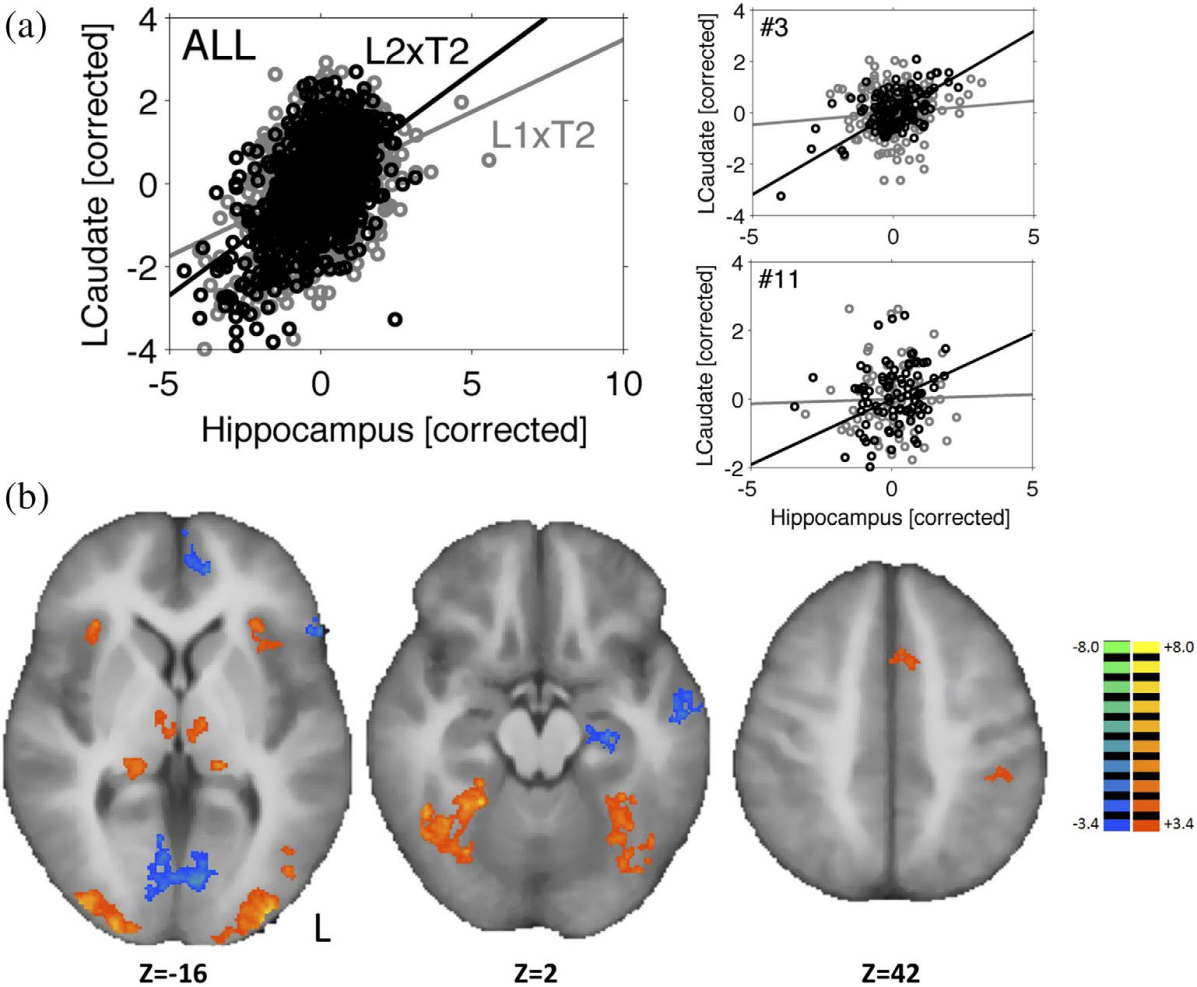
PPI scatterplots and whole‐brain results. (a) Psycho‐physiological interaction (PPI) scatterplots for activity of left caudate as a function of activity in left hippocampus for L1×T2 (grey) and L2×T2 (black) of all participants combined (ALL) as well as two representative participants (#3 and #11). (b) Areas of significantly (cluster‐level corrected) increased (hot colors) or decreased (cool colors) activity for the task, that is, L1×T2 + L2×T2. Left hemisphere shown on the right in each panel [Color figure can be viewed at wileyonlinelibrary.com]

Figure 4 shows the scatterplots of the normalized fMRI signal of the left caudate as a function of fMRI signal in left hippocampus (average of the three significant subfields). The different regression lines, showing a higher correlation for the L2×T2 condition (black line) compared to the L1×T2 condition (grey line), indicate that the PPI interaction term represented increased hippocampal‐striatal connectivity when the tested temporal interval matched the learned interval.

### 3.4 Whole‐brain analysis

Finally, we conducted a more explorative whole‐brain voxel‐by‐voxel analysis of the main effect of the task, that is, L1×T2 + L2×T2. Voxel‐level results were initially thresheld at an uncorrected *p* value of .005 and were further controlled for multiple comparisons at the cluster‐level at a false positive rate of .05. Results (see Figure 4) showed increased activity during trials of both conditions in bilateral lateral occipital cortex, inferior temporal cortex, supplementary motor area, left sensorimotor cortex and medial thalamic and lateral geniculate nuclei. Decreased activity was found in left hippocampus, ventral medial prefrontal cortex, posterior parietal cortex and medial occipital cortex at putative primary visual cortex. The contrast of L1×T2–L2×T2, corrected for multiple comparisons at the cluster‐level, revealed no significant effects.

## 4 DISCUSSION

We utilized UHF 7 T fMRI to examine how memory‐based temporal expectation is represented in the hippocampus and DS. Significantly lower activations were detected in several hippocampal subfields, including bilateral CA1 and left DG and CA3/CA2, when temporal context at retrieval matched the context used during learning (L2×T2), compared to when it did not match (L1×T2). The involvement of CA3–CA1 subfields in our task fits with previous neurophysiological studies that showed the involvement of CA subfields in processing temporal associative context. Rat lesion studies have identified CA3, CA2, and CA1, rather than DG, as critical subfields for the formation of associations between objects separated by a temporal delay (Farovik et al., 2010; Hunsaker & Kesner, 2008; Hunsaker, Thorup, Welch, & Kesner, 2006). Moreover, the presence of “time cells” that represent the temporal moments of events have been observed in CA3 (Salz et al., 2016) as well as CA2 and CA1 (Kraus, Robinson, White, Eichenbaum, & Hasselmo, 2013; MacDonald et al., 2011, 2013). However, there is some evidence that DG codes temporal contexts at various time scales, which may be related to neurogenesis (Rangel et al., 2014). Further, hippocampal activity in humans has also been associated with the encoding of temporal context (DuBrow & Davachi, 2014; Ezzyat & Davachi, 2014; Hsieh, Gruber, Jenkins, & Ranganath, 2014; Montchal, Reagh, & Yassa, 2019; Schapiro et al., 2012). Interestingly, we found that left rather than right hippocampus was associated to differences in temporal expectancy. Some previous studies showed the largest activity changes in the left hippocampus (DuBrow & Davachi, 2014; Ezzyat & Davachi, 2014) while others found activity in right (Thavabalasingam et al., 2019) or bilateral hippocampi (Hsieh et al., 2014; Schapiro et al., 2012). This heterogeneity in results may be due to task conditions, which could bias processing of some temporal contextual features over others. In our study, the ordering of cue‐target pairs was kept constant while the temporal gap between cue and target could vary, suggesting that left hippocampus may be involved in processing of temporal duration. However, other studies showed involvement of bilateral or right hippocampus when duration between items in a sequence was varied (Thavabalasingam et al., 2018, 2019). In addition, left hippocampus activity is also modulated by contextual boundaries that segment a series of items into distinct sequences (Ezzyat & Davachi, 2014; Hsieh et al., 2014), which could indicate that, in our task, duration was related to temporal expectancy of the boundary event that marked the end of a sequence (i.e., second item of a pair).

We also found increased signal amplitude in the right putamen when temporal expectations were violated. Generally, this finding fits with the long‐held hypothesis that DS is involved in processing of timed stimulus‐response gaps (Buhusi & Meck, 2005; Coull et al., 2004; Rao et al., 2001; Tanaka et al., 2004; van Rijn et al., 2014; Wiener, Turkeltaub, & Coslett, 2010), but also extends it to processing of temporal information in associative memory. Particularly, our finding mirrors a previous finding of right putamen activity when temporal expectancy about reward delivery was violated (Mcclure et al., 2003). Thus, in our study, right putamen may have coded for the violation of temporal expectation that arose from associative memory. This further fits with the more general notion that DS monitors the difference between temporal expectancies and experiences, possibly to optimize future action selection (Seymour et al., 2004).

Importantly, our PPI analysis showed increased functional connectivity between left hippocampus and striatum when temporal context during testing matched the learned temporal context. This finding is in line with reports that hippocampus and striatum interact cooperatively in the processing of associative memories (Mattfeld & Stark, 2015; Scimeca & Badre, 2012). Previous studies have shown that the two subcortical structures interact during spatial navigation of learning of relevant locations in space (Gengler et al., 2005; Igloi et al., 2010; Voermans et al., 2004; Woolley et al., 2015). Our findings extend this notion to learning of temporal associations. Interestingly, one study reported increased hippocampal‐striatal connectivity when durations within a series of items did not match those that were previously encoded (Barnett et al., 2014). Further, this study reported decreased hippocampal activity when encoding and testing durations differed, in contrast to our study, which suggests that hippocampal‐striatal connectivity may depend on the behavioral goals or outcomes of temporal processing. This may be reflected in the use of different tasks, in which participants made explicit temporal judgments in the previous study, while in our study the temporal differences were implicit.

Some final remarks about our study are warranted. The sample size, although arguably small, is comparable with other recent 7 T imaging studies (e.g., [Ten Oever et al., 2016; Protopapa et al., 2019]). We optimized statistical power by restricting the analysis search space to a small set of task conditions and subcortical regions‐of‐interest. A possible limitation of the study, however, is that general performance accuracy was lower than that in a previous study, in which participants were not scanned (van de Ven et al., 2017). This difference in performance suggests that the scanner environment may have affected task performance. Finally, the trend for lower accuracy in L1 compared to L2 trials during retrieval may limit interpretation, as it could suggest that our findings resulted from differences in expected time delays as well as a difference in memory strength. Our control analyses revealed no evidence for the latter scenario. Further, we mitigated the risk for a confounding effect of differences in memory strength by including only correct trials for the fMRI analysis. Thus, we suggest that an interpretation of our data in terms of (violation of) temporal expectation is more viable than an interpretation in terms of differences in overall memory strength, although we acknowledge that a role of memory strength cannot be entirely ruled out.

In conclusion, our fMRI study demonstrated that elapsed time in associative memory could function as an important mnemonic context for the hippocampus and striatum at retrieval. Our findings revealed left hippocampal subfields and DS as important neural correlates involved in processing temporal information of memory. Moreover, these regions were functionally connected when the temporal context at retrieval was identical to the learned temporal context. The results extend current knowledge of memory and time beyond hippocampal areas and start to explain how contextual information engrained in memory is perceived and analyzed in the human brain.

## CONFLICT OF INTEREST

All authors report no conflict of interest.

## Data Availability

The data that support the findings of this study are available from the corresponding author upon reasonable request.
